# Effects of Calendula Officinalis and Hypericum Perforatum on Antioxidant, Anti-Inflammatory, and Histopathology Indices of Induced Periodontitis in Male Rats

**DOI:** 10.30476/DENTJODS.2020.83660.1056

**Published:** 2020-12

**Authors:** Nader Tanideh, Vajiheh Ghafari, Reyhaneh Ebrahimi, Raha Habibagahi, Omid Koohi-Hosseinabadi, Aida Iraji

**Affiliations:** 1 Stem Cells Technology Research Center and Dept. of Pharmacology, School of Medicine, Shiraz University of Medical Sciences, Shiraz, Iran; 2 Students Research Committee, School of Dentistry, International Branch, Shiraz, University of Medical Sciences, Shiraz, Iran; 3 Dept. of Periodontology, School of Dentistry, Shiraz University of Medical Sciences, Shiraz, Iran; 4 Dept. of Biomaterials, Orthodontic Research Center, School of Dentistry, Shiraz University of Medical Sciences, Shiraz, Iran; 5 Laboratory Animal Centre, Shiraz University of Medical Sciences, Shiraz, Iran; 6 Central Research Laboratory, Shiraz University of Medical Sciences, Shiraz, Iran

**Keywords:** Calendula officinalis, Hypericum perforatum, Periodontitis, Rats

## Abstract

**Statement of the Problem:**

Periodontitis is one of the most common bacterial infections of the oral cavity. It is important to find adjunctive methods for chemical treatment of periodontitis with less complications and proven therapeutic properties.

**Purpose:**

The aim of this study was to compare the effects of *Calendula officinalis* and *Hypericum perforatum* on antioxidant, anti-inflammatory and histopathologic indices of induced periodontitis in male rats.

**Materials and Method:**

In this experimental animal study, forty adult male Sprague-Dawley rats were randomly divided into 4 groups (n=10) and then experimental periodontitis was induced by 3-0 nylon non-absorbable ligature. Each group was treated for 10 days as follows: 1) *H. perforatum* hydroalcoholic extract, 1000 mg/kg/daily, orally; 2) *C. officinalis* hydroalcoholic extract, 1000 mg/kg/daily, orally; 3) a mix of the two plants, 1000 mg/kg/ daily, orally, 4) normal saline solution. At the end of study, blood sample were obtained via cardiocentesis, the rats were euthanized, and their maxillae were removed. The samples were analyzed for histopathological scores and total antioxidant capacity and IL-1β were measured.

**Results:**

Mixed hydroalchoholic extract of *H. perforatum* and *C. officinalis* decreased IL-1β (4.3020±0.63), and increased the antioxidant parameter in comparison to the control group (3.1192±0.43) (*p*< 0.001). There were significant histopathological differences between the treatment groups and the control group.

**Conclusion:**

Mixed hydroalchoholic extract of *H. perforatum* and *C. officinalis* might be considered as an adjunctive treatment for periodontitis.

## Introduction

Periodontal disease is a major public health problem worldwide. It is a pathological condition with detrimental effects on the toot-supporting structures and tissues and is the most important cause of tooth loss in the adult population [ 1 ]. Inflammatory cells, especially polymorphonuclear cells, release free radicals of oxygen. Therefore, periodontitis occurs because of a decrease in antioxidant capacity and increased oxidative damage. The host's inflammatory cells release several factors, which can lead to bone loss in vitro. These factors include prostaglandins, interleukin 1-α and 1-β, and tumor necrosis factor-α (TNF-α) [ 2 ].

Pain, discomfort, cosmetic problems, and tooth loss are some of the disorders associated with periodontitis. The goal of periodontal treatment is to reduce inflammation in the inflamed tissue, decrease the counts of pathogenic bacteria, and eliminate periodontal pockets. Mechanical therapy, antimicrobial drugs, and use of systemic antibiotics are some of the clinical techniques used in the treatment of periodontitis [ 3 ].

Scaling and root planing are one of the most commonly used and effective mechanical interventions for periodontitis, which result in a decrease in pocket depth, inflammation severity and an improvement in the attachment level. While mechanical treatment including scaling and surgery can eliminate microorganisms and are the main treatment way, antibiotic therapy is also considered as an adjunctive treatment. However, systemic antibiotics might be associated with complications, including drug interactions and organ damage [ 4 ]. Herbal plants have been reported to have an important role in the treatment of various diseases. As a result, it might be of great interest to use herbal medicines as an alternative to synthetic antibiotics, in association with mechanical removal of debris [ 5 ].

*Calendula officinalis* (*C. officinalis*), also known as Marigold, is an important medicinal plant in the Asteraceae family. In traditional medicine, it is used to treat fever and cancer [ 6 ] due to its antioxidant and anti-inflammatory compounds [ 7 ]. The plant is rich in various pharmaceutically active ingredients, including sterols, flavonoids, carotenoids, and glycosides [ 8 ]. Over 100 different combinations of *C. officinalis* are extracted. Quercetin, as the main ingredient in this plant, is responsible for the plant’s major anti-inflammatory and antioxidant effects [ 9 ].

*Hypericum perforatum* (*H. perforatum*), known as St. John’s wort, is an herbal medicinal plant from the family of Clusiaceae. [ 10 ]. It exhibits antibacterial and antiviral effects through partial control of the transcription factor NF-κB, and by involving some serine/ threonine kinases from the protein kinase C (PKC) family. The main ingredients responsible for pain relief in *H. perforatum* are hyperforin and hypericin.The main anti-inflammatory and antioxidant properties of this plant are attributed to its diverse components, including hypersin and pseudopyrcine and flavonoids, such as quercetin [ 11 - 13 ]. Considering the ever-increasing administration of chemical drugs/agents and their side effects, including anaphylactic reactions, opportunistic infections and bacterial resistances to commonly used antibiotics, and also because of the popularity of herbs due to their lower cost and lower complications [ 14 ], we decided to evaluate *C. officinalis* and *H. perforatum* plants with anti-inflammatory, antioxidant and antimicrobial properties as an adjunctive treatment for mechanical and chemical therapy in periodontitis.

## Materials and Method

The experiments were carried out in accordance with the guidelines laid down by the National Institute of Health (NIH) regarding the care and use of animals for experimental procedures. Ethical considerations were confirmed by the Animal Care Committee of Shiraz University of Medical Sciences (IR.SUMS.REC ethical code: 15517).

### Animal study

Forty Sprague-Dawley male rats (aged 8-10 weeks; weighing 220±20 g) were procured from the Laboratory Animals Center of Shiraz University of Medical Sciences, Shiraz, Iran, for the purpose of this interventional experimental study. All the rats were kept at standard room temperature (22±2°C), a humidity of 55±5%, ventilation of 12 times per hour and 12 hours of light/dark cycle. The animals were fed a standard pellet diet ad libitum. 

### Preparation of hydroalcoholic extracts

*C. officinalis* flowers were collected from Fars Province (southwest of Iran), shade-dried in the laboratory at an ambient temperature of 25-30°C and relative humidity and powdered. A total of 100 g of the powdered form of the plant were transferred to adequate volume of ethanol: water (70:30) solution for 72 h using the percolation method [ 14 ]. *H. perforatum* fresh plants were procured from Shiraz, Iran. To prepare the hydroalcoholic extract, the provided plants were dried for five days at room temperature and powdered using the percolation method, and then 100 g of the powdered form of the plant were transferred to ethanol: water (70:30) solution for 72 hours.

The extracts were filtered and evaporated in a rotary evaporator under reduced pressure, dried at 50°C for 72 h and stored at -20°C. Finally, the extracts were converted to a solution of 100 mg/kg to be used for daily consumption of rats [ 15 ]. Plants species were determined in the Department of Botany of Shiraz University of Medical Sciences.

### Induction of periodontitis

**Figure 1:**
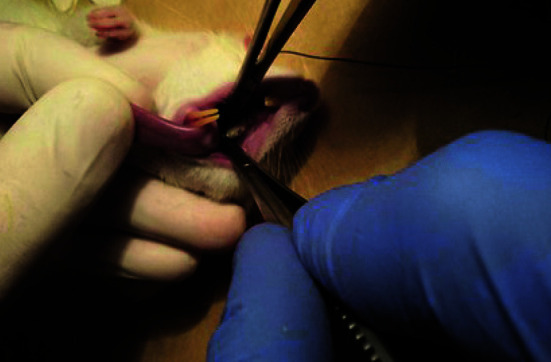
Induction of periodontitis around the second maxillary molar tooth by 3-0 nylon non-absorbable ligature in rats

The rats underwent anesthesia with 10% Ketamine hydrochloride (90 mg/kg, IM) and 2% Xylazine (5 mg/kg, IM) and nylon 3-0 non absorbable ligature (ETHIBOND EXCEL) polyester green coated braided were wrapped up around the second maxillary molar tooth of the left side and tied to the palatal area in order to induce periodontitis (Figure 1). The rats were randomly divided into 4 groups of 10, to demonstrate induction of experimental periodontitis [ 16 ].

### Grouping

- Group 1: One mL of oral solution of hydroalcoholic extract of the *C. officinalis* (1000 mg/kg/daily, orally) was fed by gavage method for 10 days daily, and on the 11th day the rats were euthanized ethically.
- Group 2: One mL of oral solution of hydroalcoholic extract of *H. perforatum* (1000 mg/kg/daily, orally) was fed orally daily by gavage method for 10 days and on the 11th day the rats were euthanized ethically.
- Group 3: A combination of *C. officinalis* and *H. perforatum*: One mL of oral solution of hydroalcoholic extract of these two plants (1000 mg/kg/daily, orally) were fed daily for 10 days by gavage method and the rats were euthanized ethically on day 11.
- Group 4: One mL of normal saline solution was fed orally daily for 10 days by gavage method and the rats were euthanized ethically on day 11. Animals were observed until the 11th day, the period of the most intense alveolar bone loss [ 16 - 17 ]. All the rats were euthanized ethically by 70% CO_2_.

Blood was prepared via cardiocentesis and serum was transferred to laboratory for inflammatory and antioxidant capacity tests. The whole maxilla was harvested and soft tissues were separated from bone and placed in 10% formalin for histopathological evaluation.

### Experimental tests

**Antioxidant activity of extracts based on 2,2-diphenyl-1-picrylhydrazyl (DPPH) assay**

The initial concentration of reagents was selected based on their absorbance close to 1.0 at measured wavelengths. As a result, freshly prepared methanolic solution of 2,2-diphenyl-1-picrylhydrazyl (DPPH) with a concentration of 0.110 mM was used to assess the radical scavenging activity of *C. officinalis* extract, *H. perforatum* extract, and equal concentration of *C. officinalis* and *H. perforatum*. Briefly, 20 μL of each sample with different concentrations (1.25, 2.50, 5, 10, 20, 40, 80, 160, 320 and 640 ng/mL) were mixed with 180 μL of DPPH reagent in a 96-well plate at 25°C. After 30 minutes of incubation in the dark, absorbance measurements were carried out in a Perkin-Elmer spectrometer at a wavelength of 517 nm. All the measurements were performed in triplicate [ 18 ].

### Antioxidant activity of extracts based on ferric reducing antioxidant power (FRAP) assay

The ferric reducing antioxidant power (FRAP) assay was carried out according to Benzie and Strain (1996) with some modifications. Briefly, the FRAP reagent contained 300 mM acetate buffer (pH= 3.6), 10mM TPTZ (2, 4, 6-tripyridyl-s-triazine) solution in 40mM HCl, and 20 mM FeCl3·6H2O solution was prepared freshly and warmed at 37°C. Aliquots of 20μL of the plant extract diluted in methanol (at different concentrations) were mixed with 180μL of FRAP reagent [ 19 ]. The absorbance of reaction mixture was measured at 595nm after 10 minutes. 

### IL1-β Test

Five mL of blood was taken from the rats. After centrifuging at 5000r/min for 5min, the supernatant was obtained and stored at -80°C. An eBioscience kit (eBioscience, San Diego, CA) was purchased and the serum level of inflammatory factor interleukin-1β (IL-1β) was determined by enzyme linked immunosorbent assay (ELISA) [ 20 ].

### Histopathological assessments

The tissue obtained from the left side of the maxilla was placed in 10% formalin and prepared for histopathological examination in the standard fashion; the sections were stained with hematoxylin-eosin (H&amp;E) staining. The H&amp;E slides were investigated under a light microscope carefully and different magnifications in a blind manner. 

### Investigation of inflammation and alveolar bone loss

The area between the first and second molar teeth was studied at ×40 magnification. The parameters, such as infiltration of inflammatory cells, alveolar bone integrity and cementum and collagen degradation were evaluated and graded ranging from minor infiltration of inflammatory cells and no collagen degradation to severe infiltration of inflammatory cells and collagen degradation scored from 0-3 respectively. These scores were defined as score 0: absence of or only discrete cellular infiltration, few osteoclasts, preserved alveolar process and cementum; score 1: moderate cellular infiltration, presence of some osteoclasts, some but minor alveolar process resorption and intact cementum; score 2: accentuated cellular infiltration, large number of osteoclasts, accentuated degradation of the alveolar process and partial destruction of cementum; and score 3: accentuated cellular infiltrate and total destruction of alveolar process and cementum [ 21 ].

### Statistical analysis 

After assessing the normal distribution of the data with the Kolmogorov-Smirnov test, one-way ANOVA was used to make comparisons between the groups. The nonparametric equivalence-Kruskal-Wallis test was used for quantitative data. To compare qualitative factors such as histopathological scores between the different groups on different days, Mann-Whitney U test was used. A p value of ≤0.05 was considered statistically significant. All the statistical analyses were carried out by SPSS20. 

## Results

### Comparative antioxidant potential of tested extract

Based on DPPH assay, the extract of *C. officinalis* in combination with the extract of *H. perforatum* demonstrated the best antioxidant activity with IC50 of 3.07±1.82 ngr/mL, followed by *H. perforatum* extract (IC50= 25.03±4.45ngr/mL). The least antioxidant activity in these series belonged to *C. officinalis* (IC50= 328.63±14.44ngr/ mL). The IC50 of quercetin as a positive control was 9.43 (±2.26 µM). The values were expressed as mean ± standard error of mean (SEM) of triplicate experiments (Table 1).

**Table 1:** Mean, maximum, minimum, and standard error of serum total antioxidant capacity (TAC) values in different groups

Group	Minimum	Maximum	Mean±Std. Error	Interaction between groups (sig)
*C. officinalis*	1.33	2.32	1.9720±.29169	*C. officinalis* with normal group (*p*< 0.001)
*H. perforatum*	1.68	2.40	2.0136±.21963	*H. perforatum* with control group (*p*< 0.001)
Mixed group	2.47	4.16	3.1192±.43179	Mix with control group (*p*< 0.001)
Normal Saline	.79	1.75	1.1113±.28067	Mix with *H. perforatum* (*p*< 0.05)
				*C. officinalis* with *H. perforatum* (*p*> 0.05)

**Figure 2:**
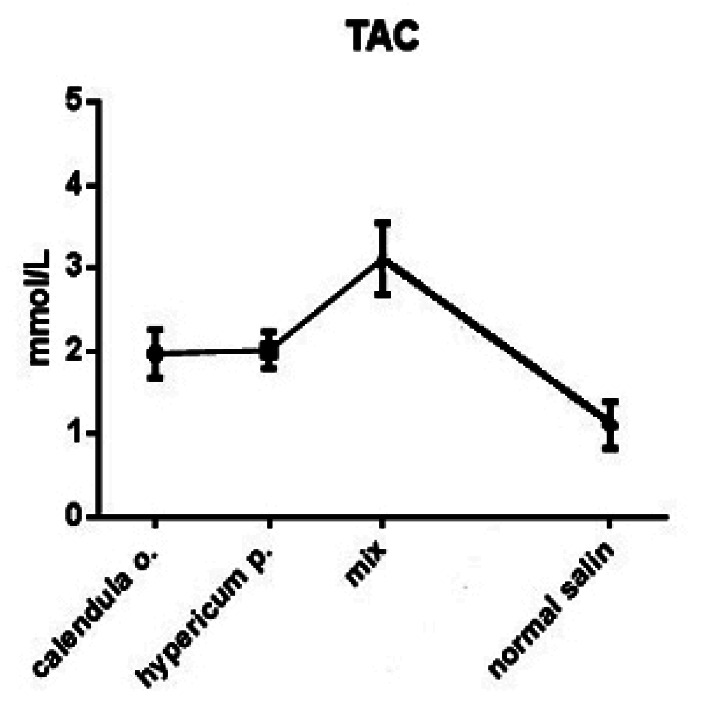
Total antioxidant activity (TAC) of *C. officinalis*, *H. perforatum*, and *C. officinalis* in combination with *H. perforatum* extract. The difference between mean and standard deviation of serum TAC index in different treatment groups is illustrated.

The principle of FRAP method is based on the reduction of a ferric-tripyridyltriazine complex to its ferrous colored form in the presence of antioxidants. The results are express as mmol ferrous ion equivalent per liter. The antioxidant activity determined by FRAP assay is reported in Figure 2. Based on FRAP technique, the combination of *C. officinalis* and *H. perforatum* showed the highest FRAP value, followed by *H. perforatum* extract and *C. officinalis*. Although all the three tested extracts demonstrated potent antioxidant activities, a combination of them can be well effective in the treatment of inflammation.

### IL-1B Test

The results of this test were presented based on the serum levels of IL-1 beta (pg/mL) (Table 2). There were significant differences between the experimental groups and the normal saline group (*p*<0.05).

**Table 2:** Mean, Maximum, Minimum and standard error of serum IL-1β values in different groups. A p Value of ≤0.05 was considered statistically significant.

Group	Minimum	Maximum	Mean±Std. error	Interaction between groups (sig)
*C. officinalis*	4.29	5.80	5.0718 ± .51285	*C. officinalis* with control group (*p*< 0.001)
*H. perforatum*	4.20	6.54	5.1933 ± .79569	*H. perforatum* with control group (*p*< 0.001)
Mixed Group	3.55	5.21	4.3020±.62512	Mix with control group (*p*< 0.001)
				Mix with *C. officinalis* (*p*> 0.05)
				Mix with *H. perforatum* (*p*> 0.05)
Normal Saline	7.29	11.53	9.1640 ±1.34356	Mix with *H. perforatum* (*p*> 0.05)
				*C. officinalis* with *H. perforatum* (*p*> 0.05)

**Figure 3:**
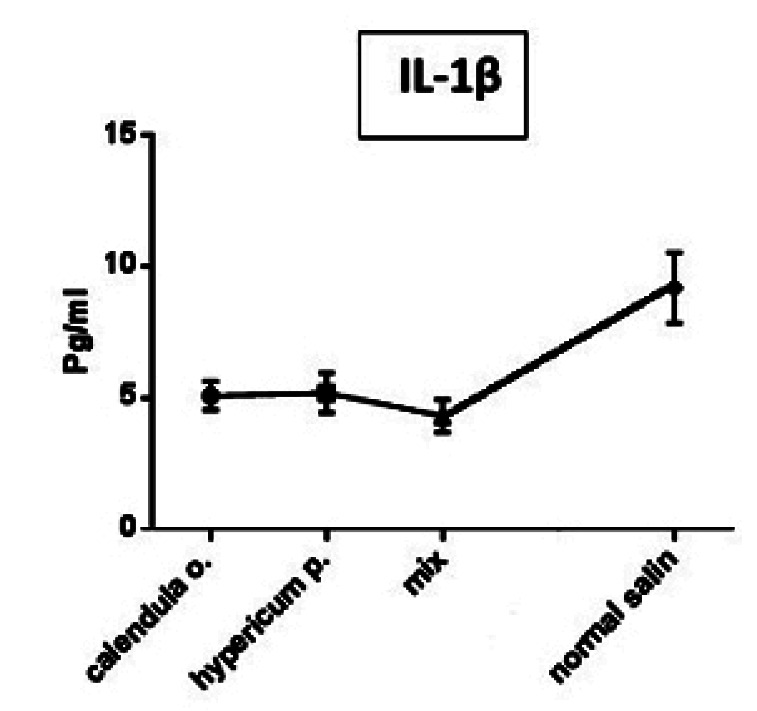
Differences between mean and standard deviation of serum IL- 1β index in different treatment groups

The lowest and highest levels were recorded in the mixed (mean= 4.302±0.62) and the normal saline groups (mean= 9.164±1.34), respectively (Figure 3).

### Histopathological evaluation

**Figure 4:**
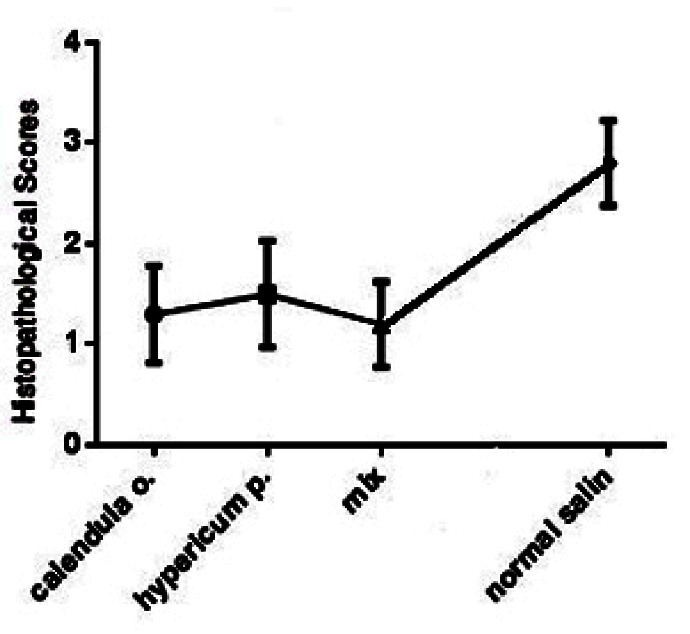
Histopathological differences between study groups and control group.

The amount of destruction in different groups was graded as follows: (0= normal, 1= mild, 2= moderate, 3= severe) and presented descriptively. Mann‒Whitney test showed statistically significant differences between the groups (*p*< 0.05) (Figure 4).

**Figure 5:**
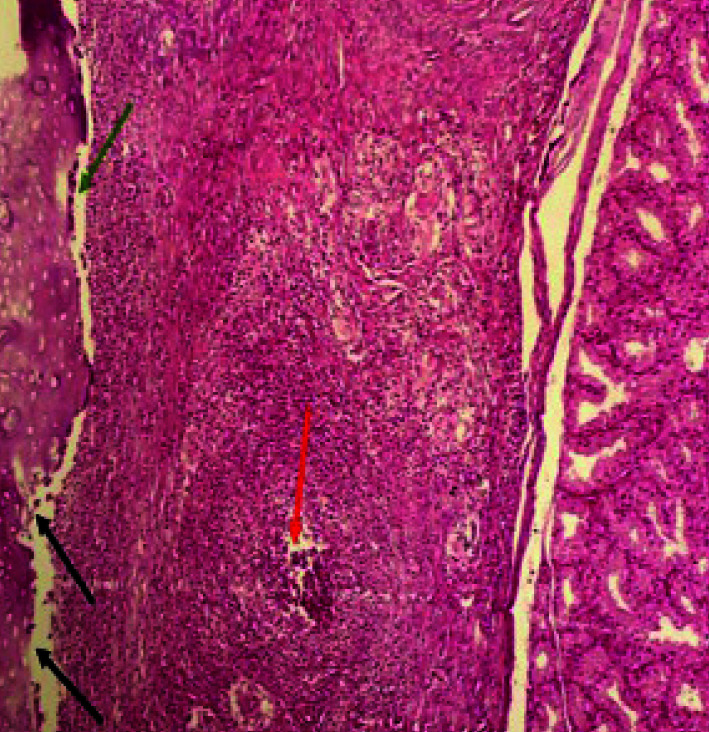
severe alveolar bone destruction (black arrows), osteoclasts during bone degradation (green arrows), severe inflammatory secretion in periodontal ligaments and formation of abscess (red arrows)

In the *C. officinalis* group, 70% were mild and 30% were moderate. In the *H. perforatum* group, 50% were mild and 50% were moderate. In the combination group, 80% were mild and 20% were moderate. In the normal saline group, 20% were moderate and 80% were severe. The highest collagen degradation and an interdental abscess in the first and second molar areas were observed in the normal saline group, and the lowest was seen in the combined group (Figure 5).

## Discussion

The results of the present study showed that use of a mixture of hydroalcoholic extracts of *C. officinalis* and *H. perforatum* for the treatment of periodontal disease resulted in a decrease in the degree of inflammation, alveolar bone loss, and oxidative stress of the tissue. IL-1β is a multifunctional cytokine and plays an active role in inflammation, immunity, and bone metabolism. Evidence shows that it promotes bone resorption, and its level is significantly correlated with periodontal attachment loss [ 22 ].

Alexandre *et al*. [ 11 ] showed that *C. officinalis* decreased the bone loss as well as the level of inflammatory mediators such as IL-1βon experimental periodontitis in rats. It was shown that the strong anti-inflammatory response of *C. officinalis* extract might be due to the inhibition of anti-inflammatory cytokines and cyclocoxygenase-2 (Cox-2) and subsequent prostaglandin synthesis. It has been reported that quercetin significantly increased osteoblast differentiation and induced mRNA expression of sialoprotein and osteocalcin in the osteoblast culture. It increased serum osteocalcin levels and the activity of alkaline phosphatase, contributing to bone tissue preservation [ 23 ].

Tanideh *et al*. [ 14 ] reported that acceleration of the healing process of mucosa in male rats treated with *C. officinalis* was significantly better than that in the control group. According to Preethi *et al*. [ 24 ], the phytochemical constituents of *C. officinalis* enhance wound healing by affecting hydroxyl proline and hexosamine contents. In addition, the anti-inflammatory effect and the antimicrobial action of *C. officinalis* have been reported to facilitate the healing of oral mucositis. Ibrahim *et al*. [ 25 ] reported significant decreases in inflammation in the rat liver by *C. officinalis* through high total antioxidant capacity of the plant. Researchers have also shown that *C. officinalis* contains large amounts of antioxidant compounds (flavonoids and polyphenols) that are responsible for their antioxidant properties; [ 9 ] these properties were shown in our study through an increase in the FRAP value of *C. officinalis* group compared to the normal saline group.

The non-enzymatic system, such as ascorbic acid, phenolics, and flavonoids as natural phyto-antioxidants, has a vital role in the development, cellular protection, and defense response against oxidative stress [ 26 ]. DPPH and FRAP are regarded as the most acceptable method to determine in vitro the antioxidant potential of the plant samples, which was one of the strengths of our study.

Collagen is the main constituent of periodontal ligament, with a key role in the architecture of periodontium. De Almeida *et al*. [ 27 ] showed that collagen breakdown is the main marker of the progression of periodontal disease. They verified that *C. officinalis* extract decreased collagen breakdown and increased collagen concentration. [ 27 ] In the present study, the lowest grade of collagen degradation was observed in the group treated with *C. officinalis* and the combined group compared to normal saline solution group.

Heijnen *et al*. [ 28 ] showed that *C. officinalis* decreased periodontal oxidative stress, which was attributed to the presence of two antioxidant pharmacophores within the quercetin molecule that have the optimal configuration for free radical scavenging. There was a positive correlation between phenolic compounds and antioxidant activity of *C. officinalis* and *C. officinalis* is rich in polyphenolic compounds.

Histopathologic and radiographic evaluation of myoperoxidase, P-selectin, and IL-1β in male rats with induced periodontitis showed that *H. perforatum* had anti-inflammatory properties and significantly decreased all the inflammatory parameters [ 29 ]. Our results also showed lower inflammation in the group treated with *H. perforatum* compared to the normal saline solution group.

NF-κB plays a key role in the regulation of many genes that are responsible for the generation of mediators or proteins in inflammation, including TNF-α, IL-1β, and iNOS. Verma *et al*. [ 30 ] demonstrated that the *H. perforatum* treatment inhibited degradation of IκB-α and significantly decreased translocation of NF-κB. We clearly confirmed a significant increase in the production of IL-1β at 10-day interval after ligation. 

Damlar *et al*. [ 31 ] showed that *H. perforatum* oil extract improved bone defects that were filled with bovine xenografts. We also had less bone loss in the *H. perforatum* group, consistent with the results of previous studies.

Osteoblasts and osteoclasts regulate bone turnover and are involved in bone formation and resorption, respectively. It had been demonstrated that *H. perforatum* extract increases MG-63 human osteoblast cell proliferation and increases bone formation by stimulating osteoblast differentiation and proliferation. It also reduces bone resorption by up regulating gene expression of osteoprotegerin (OPG) which plays an important role in bone turnover [ 32 ].

Tanideh *et al*. [ 15 ] showed that both topical and systemic forms of *H. perforatum* had significant and positive effects on mucositis. Wound-healing activity of *H. perforatum* extract seems to be mainly due to an increase in the stimulation of production of collagen by fibroblasts and the activation of fibroblast cells in polygonal shape, with a role in wound repair by closing the damaged area [ 33 ].

However, antibiotics are widely used for treating periodontitis, and their short-term effect is obvious. Nonetheless, in recent years, the resistance of pathogenic microorganisms to antibiotics has been increased due to the misuse, resulting in a decrease in the efficacy of antibiotics. Subsequently, medicinal plants with less complications and appropriate properties in reducing and controlling the periodontitis can open up new horizons for the treatment of this disease.

Nonetheless, further research is needed on herbal medicines and their doses and regimens to demonstrate their superiority over chemical antibiotics.

## Conclusion

According to the results of the present study, mixed of *C. officinalis* and *H. perforatum* plants might be employed as alternative medicine for periodontitis.
